# Does the incremental shuttle walk test require maximal effort in young obese women?

**DOI:** 10.1590/1414-431X20165229

**Published:** 2016-07-11

**Authors:** S.P. Jürgensen, R. Trimer, L. Di Thommazo-Luporini, V.Z. Dourado, J.C. Bonjorno-Junior, C.R. Oliveira, R. Arena, A. Borghi-Silva

**Affiliations:** 1 Laboratório de Fisioterapia Cardiopulmonar, Departamento de Fisioterapia, Universidade Federal de São Carlos, São Carlos, SP, Brasil; 2 Departamento de Ciências do Movimento Humano, Universidade Federal de São Paulo, São Paulo, SP, Brasil; 3 Departamento de Medicina, Centro de Ciências Biológicas e da Saúde, Universidade Federal de São Carlos, São Carlos, SP, Brasil; 4 Department of Physical Therapy and Integrative Physiology Laboratory, College of Applied Health Sciences, University of Illinois, Chicago, IL, USA

**Keywords:** Incremental shuttle walk test, Exercise, Obesity

## Abstract

Obesity is a chronic disease with a multifaceted treatment approach that includes nutritional counseling, structured exercise training, and increased daily physical activity. Increased body mass elicits higher cardiovascular, ventilatory and metabolic demands to varying degrees during exercise. With functional capacity assessment, this variability can be evaluated so individualized guidance for exercise training and daily physical activity can be provided. The aim of the present study was to compare cardiovascular, ventilatory and metabolic responses obtained during a symptom-limited cardiopulmonary exercise test (CPX) on a treadmill to responses obtained by the incremental shuttle walk test (ISWT) in obese women and to propose a peak oxygen consumption (VO_2_) prediction equation through variables obtained during the ISWT. Forty obese women (BMI ≥30 kg/m^2^) performed one treadmill CPX and two ISWTs. Heart rate (HR), arterial blood pressure (ABP) and perceived exertion by the Borg scale were measured at rest, during each stage of the exercise protocol, and throughout the recovery period. The predicted maximal heart rate (HRmax) was calculated (210 – age in years) (16) and compared to the HR response during the CPX. Peak VO_2_ obtained during CPX correlated significantly (P<0.05) with ISWT peak VO_2_ (r=0.79) as well as ISWT distance (r=0.65). The predictive model for CPX peak VO_2_, using age and ISWT distance explained 67% of the variability. The current study indicates the ISWT may be used to predict aerobic capacity in obese women when CPX is not a viable option.

## Introduction

Obesity is associated with a significantly increased risk for the development of one or more non-communicable diseases and premature mortality. The incidence and prevalence of obesity has been increasing globally, including Brazil (1). The Word Health Organization (WHO) has characterized obesity as a pandemic with significant negative implications for global health (2). In Brazil, the majority of the population is overweight and obesity is the most prevalent in women(3). Obesity is a complex condition with a multifactorial etiology (2) and an elaborate treatment approach. Nutritional counseling, exercise prescription and increase of daily physical activity are central lifestyle components in the treatment of obesity. In this context, prescribing exercise and physical activity guidelines at appropriate and individually tailored levels of intensity is ideal; the ability to do this in a broadly applicable way is particularly advantageous.

Cardiopulmonary exercise testing (CPX) is considered the gold-standard for evaluation of aerobic performance (4). However, CPX requires specialized expertise and equipment that is not always readily available. Alternatively, field tests have been increasingly used in clinical practice as a surrogate to CPX when resources are limited. As an example, the incremental shuttle walk test (ISWT) has proven to be a valid and reliable functional assessment tool in the obese population (5). The ISWT is a symptom-limited assessment, with an increase of workload each minute. The test does not require a treadmill or ergometer and has demonstrated a good correlation with peak oxygen consumption (VO_2_) (5–7).

Currently, comprehensive comparisons of the physiologic responses between the CPX and the ISWT in obese individuals are limited. The aims of the present study were to: 1) compare cardiovascular, ventilatory and metabolic responses obtained during the CPX and the ISWT, and 2) develop a predictive equation to estimate peak VO_2_ based on the ISWT cardio-metabolic response in an obese female cohort.

## Material and Methods

### Design

This was an observational, cross-sectional and comparative study, and followed the STROBE statement (8). The sample size was estimated based on the relationship between the number of variables (e.g., age, height, weight, and distance) entered into the multiple regression analysis and the minimum number of observations required, with an alpha error of 0.05 and a beta error of 0.20 (9). Thus, the minimum sample size for two predictors was calculated to be 20 volunteers.

### Subjects

Females were recruited through newspaper advertisements, internet announcements and flyers from January 2011 to March 2012. Volunteers were informed about the study procedures and possible risks and subsequently signed an informed consent form approved by the Ethics Committee in Research of the Universidade Federal de São Carlos (#088/2010), São Carlos, SP, Brazil.

Women between 18 and 45 years of age, necessarily in their reproductive years, with a body mass index (BMI) greater than 30 kg/m^2^ were included in this study (1). Exclusion criteria were: 1) pregnancy; 2) active tobacco use, or abstinence from tobacco use less than 1 year prior to study initiation; 3) alcohol or drug addiction; 4) diagnosis of diabetes; 5) uncontrolled arterial hypertension; 6) other cardiopulmonary diseases, such as chronic obstructive pulmonary disease; 7) neurological and orthopedic dysfunction, which impacted the ability to perform a maximal treadmill test; 8) use of β-blockers; 9) absence of menstrual cycle.

### General procedures

All experimental procedures were performed at the Cardiopulmonary Physiotherapy Laboratory of the Universidade Federal de São Carlos. All assessments occurred during the same period of the day to avoid the influence of circadian changes, in a room with temperatures between 22–24°C and relative air humidity between 40–60%. Subjects were guided through the experimental protocols and instructed to abstain from caffeine, stimulants, and alcoholic beverages. They were also asked not to perform strenuous physical activities during the 24-h period preceding the tests, to have a good night's sleep, and to ingest a light meal at least 2 h prior to the exercise tests.

The experimental procedures were performed over 3 days. The first day consisted of anamnesis, completion of a questionnaire for the classification of physical activity level (10), anthropometric evaluation, bioelectrical impedance analysis (BIA) and pulmonary function testing by spirometry. Subjects underwent a CPX during the second day of assessment. The third visit occurred after a minimal rest interval of 48 hours, when two ISWTs were performed.

### Clinical and physiotherapeutic evaluations

Regular physical activity patterns through one's occupation, sports activities, and leisure habits were assessed through the modified Baecke questionnaire for epidemiological studies, which has been previously validated in Portuguese (11).

Body weight and height were measured with a standard scale and a stadiometer (Welmy R-110, Brazil) to the nearest 1 mm and 0.1 kg, respectively. All measurements were performed with the subjects barefoot and lightly clothed. BMI was calculated as weight in kilograms divided by height in squared meters (kg/m^2^) and the subjects were classified according to obesity level.

Evaluation of body composition by BIA (Tanita Corporation, IRONMAN, body composition monitor, model: BC-558, Japan) required complete fasting for 4 hours, bathing clothes, no shoes or any type of metal in contact with the volunteers' body, and complete elimination of urine prior to measurement. The test was performed as previously described and validated (12), and provides percent body fat (%fat), basal metabolic rate (BMR) and fat free mass (FFM) in kg.

Pulmonary function testing was performed using a mobile respiratory gas analysis system (Oxycon Mobile^®^, Mijnhardt/Jäger, Germany). The volunteers completed at least three acceptable maximal forced and slow expiratory maneuvers according to the recommendations of the American Thoracic Society (13), and the predicted values were considered in accordance with reference values for the Brazilian population (14).

### Cardiopulmonary exercise testing (CPX)

The symptom-limited CPX was performed on a treadmill (Master ATL, Inbramed, Brazil). The exercise test consisted of: 1) a 4-min rest period on the treadmill; 2) an incremental phase according to the Bruce protocol (15), and 3) a 3-min recovery period. The Bruce protocol was employed in the current study as it has been commonly used in previous studies assessing aerobic capacity in obese cohorts. During the test, all the volunteers were actively encouraged by the investigators to exert themselves maximally.

Heart rate (HR), arterial blood pressure (ABP, measured using a standard cuff sphygmomanometer) and perceived exertion by the Borg scale (16) were measured at rest, during each stage of the exercise protocol, and throughout the recovery period. The predicted maximal heart rate (HRmax) was calculated (210 – age in years) (17) and compared to the HR response during the CPX.

Before, during the test and 6 min into recovery, the volunteers were monitored by a 12-lead electrocardiogram (ECG) and portable metabolic system calibrated prior to each test (18) (Oxycon Mobile). One qualified physiotherapist with a physician supervision conducted each exercise test. Test termination criteria included: 1) volitional fatigue; 2) abnormal ABP response to exercise; 3) ECG abnormalities (i.e., ≥2 mm ST-segment depression and/or ventricular arrhythmias) (19); 4) attainment of HRmax; 5) attainment of a respiratory exchange ratio >1.20 or a plateau in VO_2_ with increasing workload.

### Incremental shuttle walk test (ISWT)

The volunteers were instructed to walk around an indoor 10-m corridor, as described by Singh et al. (6). The walking speed was dictated by a prerecorded acoustic signal on a compact disc digital audio; at the end of every minute, there was an additional signal indicating the moment at which the walking speed should increase (0.17 m/s every minute). The subjects were advised to increase speed each minute during 12 stages. Two ISWTs were performed with a 30 min interval between each, to reduce the possibility of a learning effect. Only the second ISWT values were considered for analysis (5).

The test was terminated either by the subject’s request, based on inability to continue the exercise (for example: chest pain, dyspnea, leg fatigue), or by the physiotherapist conducting the test, based on the individual inability to maintain the required speed to complete the course (0.5 m away from the cone). Chest pain, intolerable dyspnea, dizziness, leg cramps, diaphoresis and pallor were carefully monitored during the test. The same portable metabolic system (Oxycon Mobile) was used for ventilatory expired gas measurements during all ISWTs.

### Statistical analysis

Statistical analysis was performed using SigmaStat statistical software, version 2.03 (SPSS Inc., USA). Data are reported as means±SD. The following tests were carried out: 1) Kolmogorov-Smirnov to evaluate the normality of variable distribution; 2) Pearson's or Spearman's coefficient to evaluate correlation between variables [perfect correlation (r=1), strong (r>0.75), moderate (r>0.5), weak (r<0.5) and nonexistent (r=0)]; 3) paired *t*-tests to compare differences in numerical data between tests; 4) multiple regression analysis to evaluate the prediction of peak VO_2_ obtained during CPX; age and distance of the ISWT were identified a priori as predictors of peak VO_2_ during CPX. The level of statistical significance was set at 5% for all tests.

## Results

### Sample characteristics

**Figure 1:**
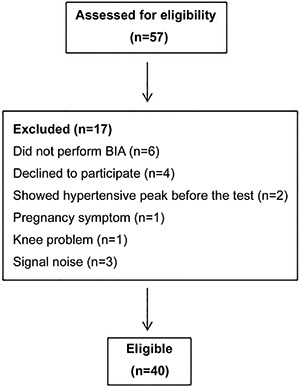
Flowchart of the subjects. BIA: bioelectrical impedance analysis.

Forty obese women were included in this study (Figure 1). Key baseline characteristics are presented in Table 1. Fifty-four percent of the subjects had level I obesity (30.0–34.9 kg/m^2^), 35% had level II (35.0–39.9 kg/m^2^) and 11% had level III (>40.0 kg/m^2^). Eight women presented with controlled hypertension; all other subjects presented with normal values for systolic and diastolic blood pressure at rest.

**Table:**
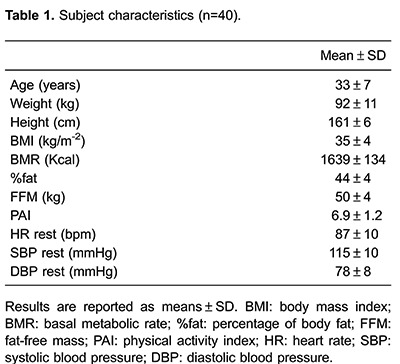

### Exercise test results

Table 2 lists exercise test results from the second ISWT and CPX. Although the maximum speed achieved in both tests was similar, the exercise response to CPX was higher when compared to the ISWT in cardiovascular, pulmonary and metabolic aspects in the studied population.

**Table:**
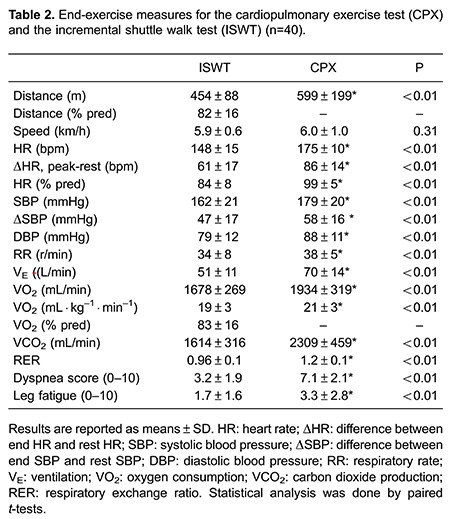

**Figure 2:**
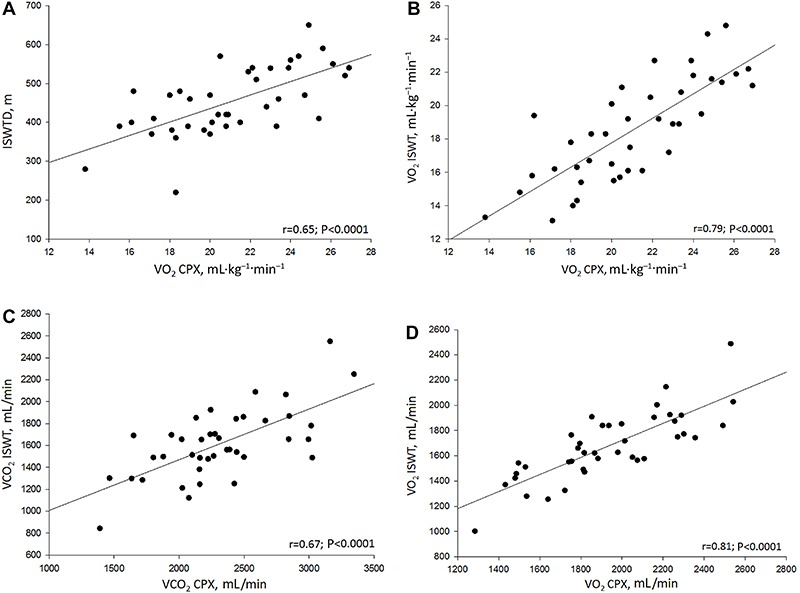
Pearson's and Spearman's correlation coefficients. *A*, correlation between ISWT distance and oxygen consumption in CPX in mL·kg^-1^·min^-1^; *B*, correlation between oxygen consumption in ISWT and CPX in mL· kg^-1^·min^-1^; *C*, correlation between carbon dioxide production in ISWT and CPX in mL/min; *D*, correlation between oxygen consumption in ISWT and CPX in mL/min. ISWT: incremental shuttle walk test; CPX: cardiopulmonary exercise testing; VO_2_: oxygen consumption; VCO_2_: carbon dioxide production.

Although submaximal effort was achieved, the variables obtained by the ISWT were correlated with the principal variables obtained during the CPX (Figure 2). There was a strong correlation between peak VO_2_ (relative and absolute values, B and D) obtained with CPX and ISWT. We also found a moderate correlation between the VCO_2_ measured by the two exercise tests, (C) as well as between the ISWT distance and peak VO_2_ during CPX (A).

### Predictor equation

Multiple linear regression for relative peak VO_2_ prediction was elaborated with the variables that presented the strongest correlations (Table 3). Both ISWT distance and age were significant predictors of peak VO_2_ obtained during CPX, and explained 67% of the variance in peak VO_2_.

**Table:**
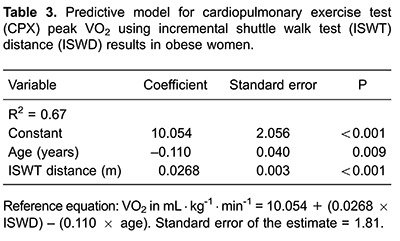

## Discussion

The main results of this study may be summarized as follows: 1) there was a significant correlation between peak VO_2_ obtained by ISWT and CPX in obese women; 2) a significant predictive model for CPX peak VO_2_, using age and ISWT distance, was found using the cohort included in this study.

Exercise prescription and increase of daily physical activity are important components in the treatment of obesity, along with nutritional counseling. In this context, the assessment of functional capacity to prescribe exercise at an ideal intensity can be a challenge in some settings. In our study, we chose to maintain a degree of sample homogeneity, selecting only adult women within reproductive age range. Therefore, the majority of subjects presented low score on the Baecke questionnaire (10), indicating the group was primarily sedentary and had similar activity patterns.

Cardiopulmonary exercise testing is the gold standard approach for assessing aerobic and functional capacity. This technique, however, requires specialized equipment and qualified professionals to conduct the test, which elevates the cost of evaluation and limits broad availability. In contrast, the ISWT has broad clinical potential, since it has been developed as a field test able to evaluate functional capacity in an incremental nature (6). The ISWT was recently validated in obese women (5).

Considering that the ISWT is a submaximal assessment, it has the potential to elicit lower cardiopulmonary stress results when compared to CPX in distinct populations (20,21). Our results are consistent with this premise as the cardiovascular profile during exercise, represented by peak HR, respiratory rate (RR) and ABP, were lower during the ISWT when compared to CPX. Additionally, peak fatigue and dyspnea scores were lower during the ISWT. These findings indicate lower physiologic stress during the ISWT, which is advantageous within the context of minimizing adverse event risk, particularly when monitoring capabilities (i.e., continuous ECG and frequent ABP measurement) are not possible. Interestingly, although the ISWT produced a lower physiologic stress we found a significant correlation with VO_2_ measured by CPX, indicating that this field test may have the ability to predict aerobic capacity, which should be explored further.

During the ISWT, the subjects covered a shorter distance (454±88 m) when compared to CPX, which moderately correlated with peak VO_2_. Collectively, the results from the current study in conjunction with previous studies demonstrate the clinical value of the ISWT in assessing aerobic capacity in the obese population. Evans et al. (22) compared the cardiopulmonary responses of both the ISWT and 6-min walk test (6MWT) with CPX results in an obese cohort (men and women with a mean BMI of 33.3 kg/m^2^, similar to our study) with obstructive sleep apnea and found a stronger relationship (r=0.87, P<0.001) between ISWT distance and peak VO_2_ during CPX compared to our findings. Oliver et al. (23) compared the metabolic responses during the ISWT and 6MWT in obese patients prior to bariatric surgery (BMI: 43.5 kg/m^2^). The authors found that the ISWT induced a higher metabolic demand compared to the 6MWT. However, as with the current study, mean peak RR values during the ISWT was <1.0, demonstrating the submaximal nature of this assessment. The lower distance covered (406 m) during the ISWT in the study by Oliver et al. (23) compared to the current study is likely due to the significantly higher BMI of participants in the former study. Previous researches in conjunction with the findings of the current study demonstrate the clinical utility of the ISWT in the obese population.

In our study, the correlations between relative and absolute VO_2_ were strong, even with a low perceived effort reported in the Borg scale at the peak of the ISWT; these results are in accordance with Evans et al. (22). The VO_2_ analysis is important for clinical practice, once it reflects the cardiopulmonary fitness. Furthermore, ISWT was able to adequately evaluate the cardiopulmonary capacity in obese women, lowering the risk for adverse effects and physical stress (22,24) compared with the use of the CPX, since running is not allowed in this test (25).

Our findings also suggest that a peak VO_2_ prediction equation by means of simple variables obtained from ISWT (age and covered distance) in obese women, might have potential validity. Our results confirm that the use of a functional test is easier and less expensive than CPX for evaluating physical fitness. Aerobic capacity is clearly an important marker of health and prognosis in apparently healthy individuals and virtually every patient population (26). As such, finding broadly applicable techniques to accurately assess aerobic capacity is of high value.

In this way, a simple field test can be used to assess physical capacity when we aim to prescribe the correct exercise intensity and reach treatment success, particularly in obesity care centers. The ISWT is a safe, rapid and inexpensive assessment that has been applied in the health care system in different populations (27–29).

A number of previous investigations (30–33) have introduced aerobic capacity prediction equations in an attempt to estimate this vitally important health measure where resources are limited (26,34). Our results indicate that the ISWT can be used for this purpose in obese females, from protocols that do not require maximal effort. Future work using independent cohorts, should be directed towards validating the regression equations available, including the one we have proposed in the current study. These validation studies are needed to solidify the clinical utility of aerobic capacity prediction.

As with any analysis, certain limitations in the current study should be considered. First, the cohort was only composed of obese women in a reproductive age range, limiting generalization of our findings. Thus, we encourage future research in obese cohorts with different baseline characteristics to further assess the utility of the ISWT and refine/validate aerobic capacity prediction. Furthermore, the current study conducted the CPX using the Bruce protocol exclusively. Using a different CPX test protocol may produce different responses and correlations with the ISWT, which should be addressed by future research.

The current study indicated the ISWT may be used to predict aerobic capacity in obese women when CPX is not a viable option. In particular, age and ISWT distance may be key predictors of peak VO_2_ in this population.
